# Cholera Epidemic in Guinea-Bissau (2008): The Importance of “Place”

**DOI:** 10.1371/journal.pone.0019005

**Published:** 2011-05-04

**Authors:** Francisco J. Luquero, Cunhate Na Banga, Daniel Remartínez, Pedro Pablo Palma, Emanuel Baron, Rebeca F. Grais

**Affiliations:** 1 Epicentre, Paris, France; 2 European Programme for Intervention Epidemiology Training (EPIET), European Centre for Disease Prevention and Control (ECDC), Stockholm, Sweden; 3 Department of Epidemiology, Ministry of Health, Bissau, Guinea-Bissau; 4 Médicos Sin Fronteras, Barcelona, Spain; University of KwaZulu-Natal, South Africa

## Abstract

**Background:**

As resources are limited when responding to cholera outbreaks, knowledge about where to orient interventions is crucial. We describe the cholera epidemic affecting Guinea-Bissau in 2008 focusing on the geographical spread in order to guide prevention and control activities.

**Methodology/Principal Findings:**

We conducted two studies: 1) a descriptive analysis of the cholera epidemic in Guinea-Bissau focusing on its geographical spread (country level and within the capital); and 2) a cross-sectional study to measure the prevalence of houses with at least one cholera case in the most affected neighbourhood of the capital (Bairro Bandim) to detect clustering of households with cases (cluster analysis). All cholera cases attending the cholera treatment centres in Guinea-Bissau who fulfilled a modified World Health Organization clinical case definition during the epidemic were included in the descriptive study. For the cluster analysis, a sample of houses was selected from a satellite photo (Google Earth™); 140 houses (and the four closest houses) were assessed from the 2,202 identified structures. We applied K-functions and Kernel smoothing to detect clustering. We confirmed the clustering using Kulldorff's spatial scan statistic. A total of 14,222 cases and 225 deaths were reported in the country (AR = 0.94%, CFR = 1.64%). The more affected regions were Biombo, Bijagos and Bissau (the capital). Bairro Bandim was the most affected neighborhood of the capital (AR = 4.0). We found at least one case in 22.7% of the houses (95%CI: 19.5–26.2) in this neighborhood. The cluster analysis identified two areas within Bairro Bandim at highest risk: a market and an intersection where runoff accumulates waste (p<0.001).

**Conclusions/Significance:**

Our analysis allowed for the identification of the most affected regions in Guinea-Bissau during the 2008 cholera outbreak, and the most affected areas within the capital. This information was essential for making decisions on where to reinforce treatment and to guide control and prevention activities.

## Introduction

Although cholera has disappeared among the diseases affecting developed countries, it remains one of the main causes of morbidity and mortality in the poorest areas of the world [1], [2]. The burden of cholera is underestimated or non-estimated and many countries face recurrent epidemics [1], [3]. Sub-Saharan African countries are especially affected, with 95% of reported cholera cases and 98% of deaths [4]. Cholera emerges under poor hygiene and sanitary conditions; thus, the lack of basic services and disorganized urbanization in many Sub-Saharan African countries constitutes the perfect culture medium for cholera [1].

John Snow, one of the founders of modern epidemiology, showed the importance of descriptive epidemiology in cholera epidemics, emphasizing the importance of “place”, or the consideration of space, to target prevention and control activities [5]. Today, although resources and tools for mapping are available, the description of place in cholera epidemics remains poor and examples of studies using spatial technologies in the medical literature are limited [6]–[15].

The objective of this study was to describe the cholera epidemic affecting Guinea-Bissau from May 2008 to January 2009 focusing on place in order to guide prevention and control activities. We also conducted a cluster analysis to obtain more detailed information about the distribution of cases in the most affected area of the capital (Bairro Bandim) with the same aim.

## Methods

### Context

The Republic of Guinea-Bissau is one of the smallest nations in continental Africa; it is divided into 8 regions (Bafata, Biombo, Bissau, Bolama, Cacheu, Gabu, Oio, Quinara and Tombali) and the capital, Bissau, (Sector Autónomo de Bissau (SAB)). The SAB is the smallest geographical region in the country but the most densely inhabited. Around 27% of the total population of the country lives in this area. Although the country has experienced several years of stability and development since the end of the civil war in 1998, the life expectancy at birth is 47 years, and 203 children die before the age of five per 1 000 live births [16].

### Descriptive epidemiology

#### Data sources

Daily official data from the Public Health General Direction (DGS) registers was used to describe the epidemic for the entire country in terms of time and place.

For SAB, we put in place a comprehensive data collection system to describe the epidemic from 05/05/2008 to 20/10/2008. These data came from the registers of the Cholera Treatment Center (CTC) in the Hopital Nacional Simao Mendes and the five Cholera Treatment Units (CTU) set up in 5 Health Care Centers (Bandim, Bairro Militar, Ajuda, Antula and Plaque). The collected data included age, sex, place of residence (*bairro* and *area sanitaria*), center where the patient was treated, and clinical outcome (dead or alive).

Population data (denominators) used for rate estimations were obtained from the 1991 census of Guinea-Bissau. A specific growth rate was applied to the different Sanitary Areas (SA) to account for population growth and migration (provided by the Epidemiological Department of the DGS (SE-DGS)). Age and sex distributions were obtained from the annual SE-DGS census projections.

#### Case definitions, laboratory procedures and statistical analysis

A modified WHO clinical case definition was used for suspected and confirmed cases of cholera [17]. A suspected case was defined as any person suffering from acute watery diarrhea with or without vomiting. A confirmed case was defined as any suspected case with a positive stool sample to *Vibrio cholera O1* or *O139*.

Stool samples were analyzed in the National Laboratory of Microbiology (NLM) in Bissau to confirm the cholera outbreak and to determine the current circulating strain and its antibiotic sensitivity by culture [18]. Additional samples were sent to the Pasteur Laboratory in Dakar for the same purpose.

We describe the cholera outbreak in Guinea Bissau in terms of time and place. For SAB, we describe the epidemic in terms of time, place and person. Central tendency (mean and medians) and dispersion parameters (standard deviation and interquartile range) were calculated for continuous variables, percentage and 95% confidence intervals for categorical variables. To adjust attack rates by age and sex, we used a Poisson regression model.

### Survey: cluster analysis

We conducted a cluster analysis to identify areas at high risk of infection in the Bairro Bandim Health District of SAB. Bandim was selected because this neighborhood reported the highest attack rates and the most cases within SAB.

#### Sample size

To calculate the sample size, we assumed that 20% of the households would have at least one case. We aimed to detect statistical differences for areas with at least 30% of households with one case. Considering a power of 80% and an alpha error of 0.05, the required sample size was 626 households. Assuming 10% of households would be either refusals or absences, the sample size was 678 households. We randomly selected 140 houses from the 2,202 structures identified in the satellite photo obtained from Google Earth™. To do so, we assigned a number to each house. We then used a random number, using the random generator function implemented in R© Statistical Software [19], for selection. For field teams to locate houses, coordinates (WGS 84) of the randomly selected houses were introduced into a handheld GPS (Garmin XL). The team carried out an active search of cases in the selected household and in the four closest households (defined by the field teams) in order to reach the desired sample size in a reasonable time without loosing precision due to the design effect [20]. Thus, the final sample frame was 700 households.

#### Statistical analysis

Our spatial point pattern consisted of locations with at least one cholera case and houses without cases. Thus, the data represented a typical example of a marked point pattern. We analyzed whether or not the observed cholera cases were clustered over and above the level that would be expected under natural environmental heterogeneity. We calculated the K-functions for both the houses without cases and those with at least one case, and the difference was used to detect the extra propensity of the households with cases to cluster. We calculated the standard error for the difference of the K-functions and the 95% confidence intervals in order to know if the observed difference was different from zero, meaning clustering occurred [21]. Next, we computed the probability of finding a house with at least one case using a Kernel smoothing technique [22]. The significance of departure from randomness was assessed by random labeling performing 1000-time simulations to establish upper and lower confidence limits. These analyses were performed using in R© Statistical Software [19]. We confirmed the clustering using Kulldorff's spatial scan statistic using SatScan software [23]. We performed 9999 Monte Carlo replications to obtain estimates of P values and confidence intervals.

### Ethical considerations

As this study was conducted during the emergency response to the cholera outbreak and was designed to provide information to orient the public health response, ethical approval was not sought prior to the survey. We sought retrospective approval from the National Ethical Review Board (ERB) of Guinea-Bissau and from the MSF ERB. The MSF ERB considered that since the purpose of the study was to guide prevention and control activities, it could be considered good public health practice rather than research. The National ERB of Guinea-Bissau granted retrospective approval.

Privacy, confidentiality and rights of patients were ensured during and after the conduct of the study. Oral informed consent was obtained in each visited household after detailed explanation of the existence of an outbreak, the objective of study and the planed use of the information. Moreover, health education was carried out in each household regarding cholera transmission and prevention. The information was entered and analyzed anonymously. The study was implemented in collaboration with the Ministry of Health after obtaining authorization to carry out the survey.

## Results

### Descriptive epidemiology

**Figure 1:**
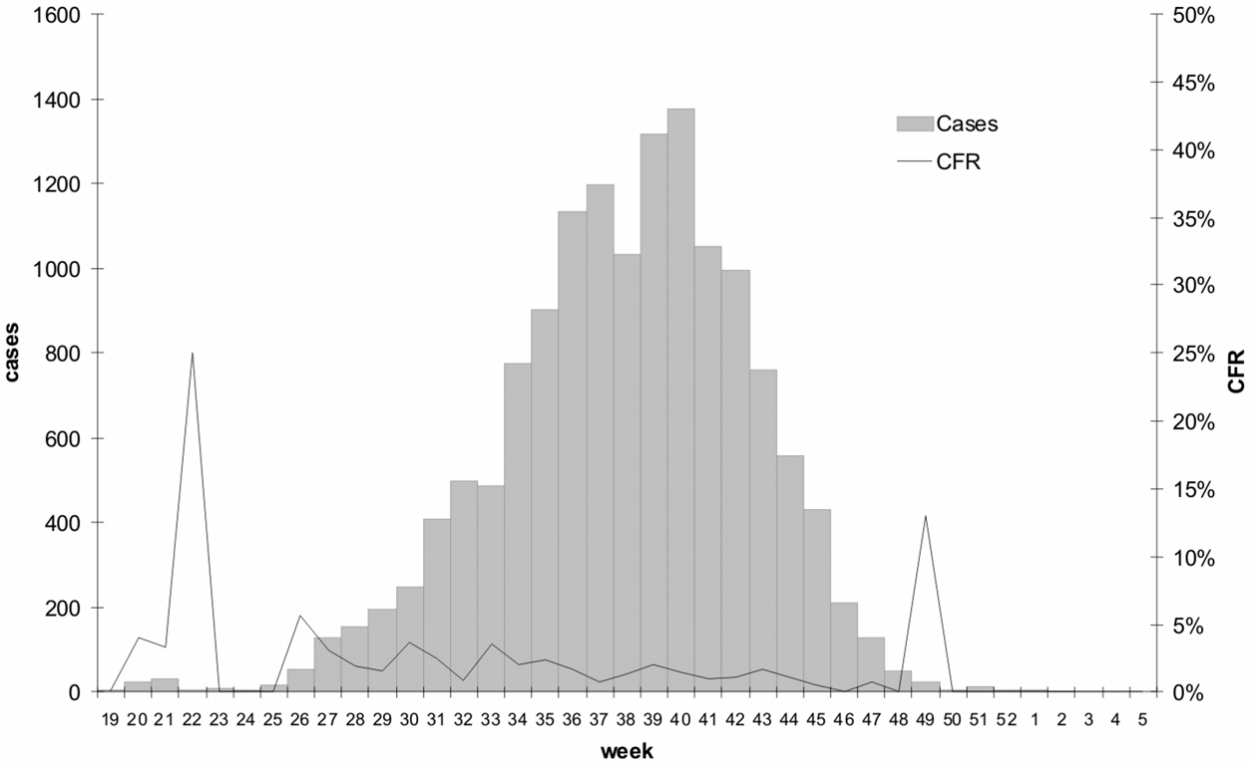
Weekly number of cholera cases and case fatality ratio (CFR%) in Guinea-Bissau 2008–2009.

The first cholera case was declared in Guinea-Bissau on 5 May 2008 (week 19). The epidemic was officially declared in July. Both the NLM and the Pasteur Laboratory in Dakar confirmed that the circulating strain was *Vibrio Cholera O1* El Tor-Ogawa. A total of 14,228 suspected cases and 225 deaths were declared in the whole country. This number of cases corresponds to an attack rate of 0.94 (AR) per 100 people. The reported case fatality ratio (CFR) per 100 cases was 1.58. The weekly average number of cases was 395.2 (SD 454.6) and the median 176.0 (IQR = 15.5–764.0). The weekly number of cases varied between a minimum of 2 cases and a maximum of 1,376 cases. The peak of the epidemic was observed after 22 weeks of reported cases, which corresponds to epidemic week 40. After the peak, fourteen consecutive weeks with decreasing numbers of cases were observed (Figure 1).

**Figure 2:**
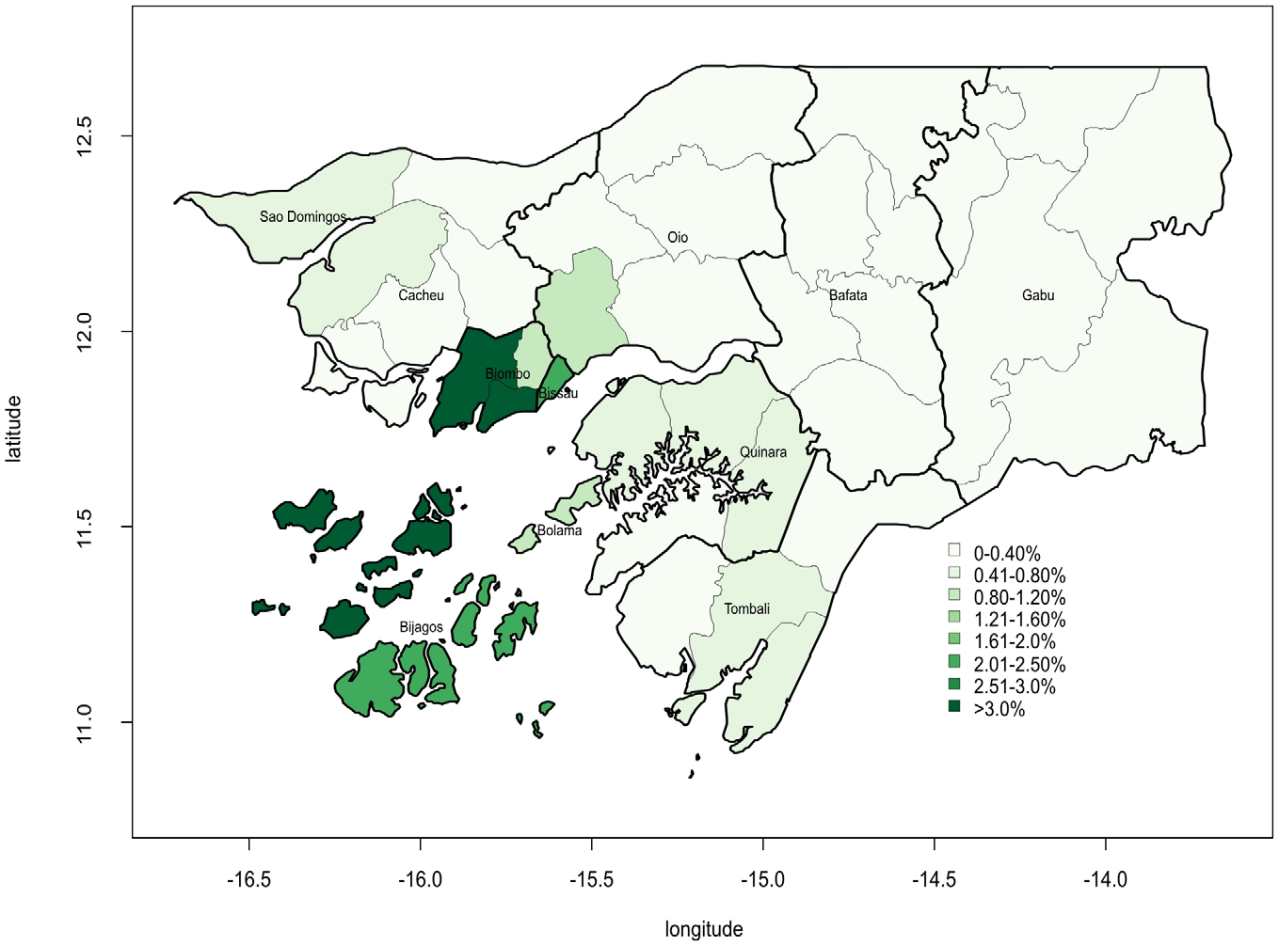
Geographical distribution of the crude cholera attack rate by region and sub-regions in Guinea- Bissau, 2008–2009. Coordinates expressed in sexagesimal degrees.

Figure 2 shows a more detailed description of the geographical distribution of the epidemic. There were several sub-regions with AR over 2% (SAB; Quinhamel and Prabis in Biombo; Bubaque and Uno in Bijagos) and two of them (Ondame in Biombo and Caravelas in Bijagos) reached 4%.

The SAB reported 67% of the total number of reported cases in the country. The first case was declared 5 weeks after the first notification in Tombali. Finally, 9,394 cases and 73 deaths were reported in the SAB, which corresponds to an AR of 2.33%. The reported CFR was 0.78%. The peak of the epidemic was observed in epidemiological week 40. Until the 20 October 2008 (period with individual data collection), a total of 7,749 cases were reported in the CTC and 5 CTUs of SAB. Most cases were treated at the CTC (68.4%, n = 5300/7749). Regarding the CTUs, the Bandim CTU received the most patients (9.8%). Most patients were between 15 and 49 years old (68.6%). This group showed the highest attack rate, almost 3.5 times higher than in the youngest age group. The number of reported cases was similar in men (48.6%) and women, with similar attack rates.

**Figure 3:**
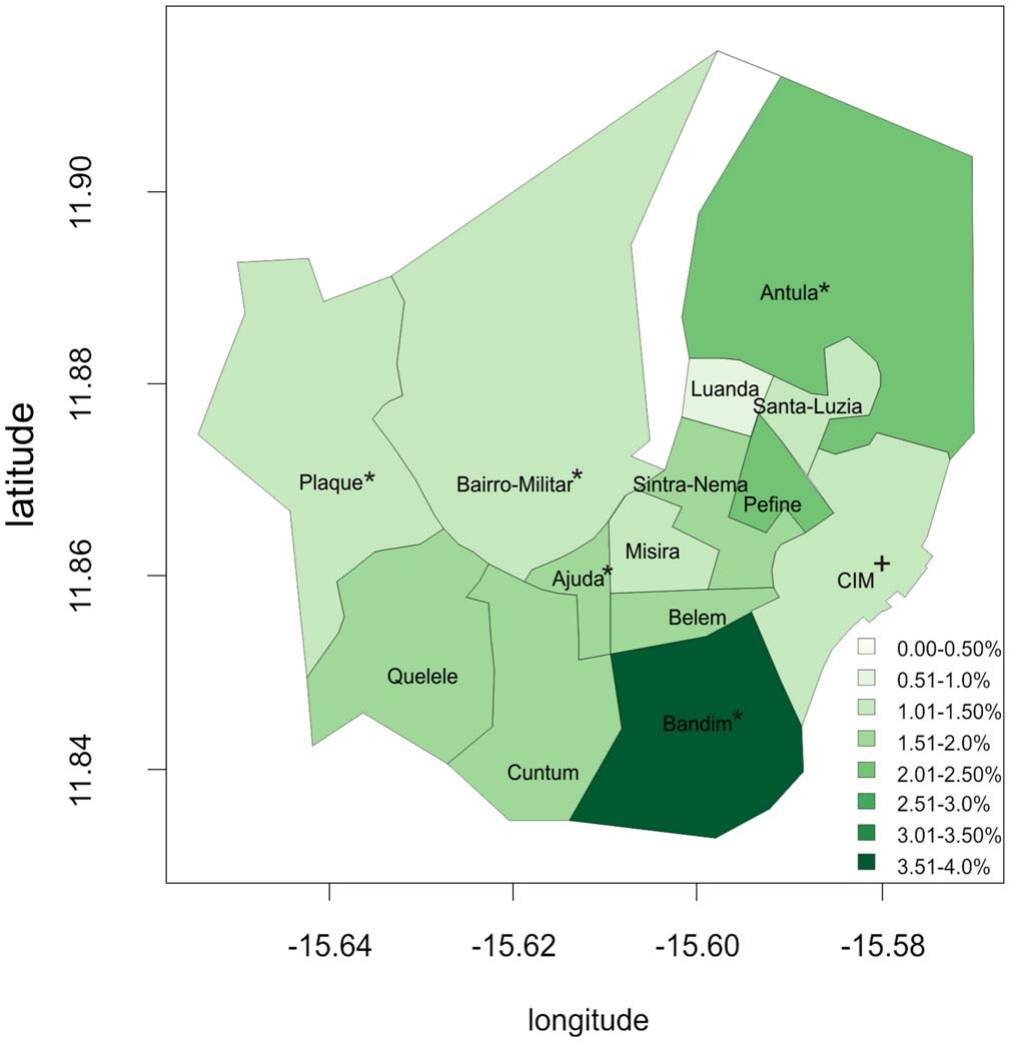
Age and gender adjusted cholera attack rates (%) by Sanitary Area in Sector Autónomo de Bissau, 2008–2009. Coordinates expressed in sexagesimal degrees. * Sanitary area with a cholera treatment centre. + Sanitary area with a cholera treatment unit.

We obtained information about the SA of residence from 7,294 patients (94.1%). Bandim was the SA with the highest number of patients in absolute numbers (24.1%) but also in relative numbers (AR = 3.9%). Compared with Ajuda, people living in Bandim were 2.5 times more affected (Table 1). There was not a clear spatial pattern in the SAB, but all neighborhoods located in the southwest had ARs over 1.5% (Figure 3).

**Table 1:** Number of cases, population, attack rate per 100 people (AR%), risk ratios (RR) and adjusted risk ratios (ARR) by age, sex and sanitary area.

Variable	Cases	Population	AR%	RR	ARR
Gender					
Female	3 960	203 946	1.94	Ref.	
Male	3 744	199 052	1.88	0.97	0.98
Age (years)					
0–14	1 632	189 409	0.86	Ref.	
15–49	5 312	174 337	3.05	3.54	3.54
>50	798	39 252	2.03	2.36	2.27
Sanitary Area					
Ajuda*	160	10 429	1.53	Ref.	
Antula*	640	30 778	2.08	1.36	1.37
Bandim*	1 756	44 718	3.93	2.56	2.61
Bairro Militar*	932	65 274	1.43	0.93	0.94
Belem	306	17 263	1.77	1.16	1.18
CIM**	154	14 985	1.03	0.67	0.68
Cuntum	857	45 482	1.88	1.23	1.24
Luanda	219	25 236	0.87	0.57	0.58
Missira	516	38 838	1.33	0.87	0.87
Pefine	306	14 808	2.07	1.35	1.37
Plaque*	380	27 633	1.38	0.90	0.89
Quelele	472	28 898	1.63	1.06	1.08
Sintra_Nema	348	21 451	1.62	1.06	1.08
Santa Luzia	248	17 204	1.44	0.94	0.96
Total	7 749	402 998	1.92	-	-

*Cholera treatment unit set up in this area.

**Cholera treatment centre set up in this area.

Sector Autónomo de Bissau, 2008.

### Cluster analysis

From the 140 structures randomly selected, we were able to assess 136. Of the 4 structures not included in the analysis, 2 were not households (one was a cinema and the other a carpentry) and 2 houses were uninhabited. As we also assessed the four closest households to those randomly selected, a total of 616 households were included in the analysis. We found at least one case in 140 households (22.7%; 95%CI: 19.5%–26.2%).

**Figure 4:**
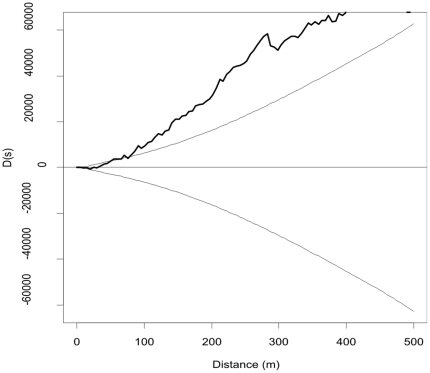
Differences of K-functions and 95% confidence intervals between households with cholera cases and households without cases in Bairro Bandim (Bissau), 2008–2009. A homogeneous set of points in the plane is a set that is distributed such that approximately the same number of points occurs in any circular region of a given area. A set of points that lacks homogeneity is spatially clustered. The k-function is defined as the expected number of points within a distance *s* of an arbitrary point, divided by the overall density of the points. Due to variations in the spatial distribution of the population at risk, a k-function computed only for cases may not be informative. Instead, the k-function calculated for cases can be compared with the one calculated for non-cases, with the difference between the two functions representing a measure of the extra-aggregation of cases over and above the observed for the non-cases. This difference is represented in the figure above, showing extra-aggregation of cases.

**Figure 5:**
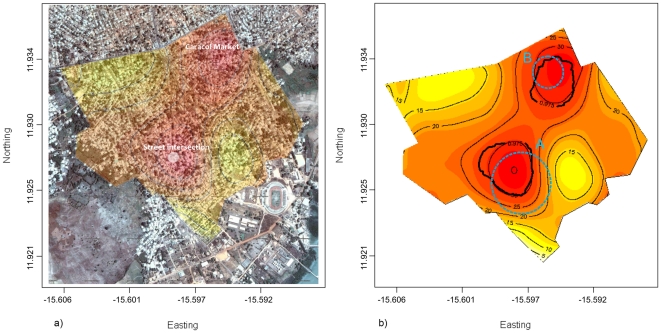
Geographical distribution of the probability of finding a house with at least one cholera case in Bairro Bandim (Bissau) in percentage and areas over the 95% confidence interval. Coordinates expressed in sexagesimal degrees. Figure 5a shows the Google Earth™ picture and the overimposed image of the risk surface (probability of finding a house with at least one cholera case). The figure 5b shows the risk surface and the two areas with statistically significant higher risk (black bold line). The same two clusters were detected (dashed blue circles) using the Kulldorff's spatial scan statistic (cluster A: Log likelihood ratio = 9.95, P = 0.029; cluster B: Log likelihood ratio = 8.81, P = 0.05).

We computed the K-functions for the 476 houses without cases and the 140 houses with at least one case. We also computed the difference between both K-functions and the 95% confidence intervals as explained previously [21]. This comparison showed that the houses with cases were more clustered than houses without cases (p<0.001) (Figure 4). Next, we computed the probability of finding a house with at least one case using a Kernel smoothing technique. Two clusters were identified in the study area using both random labeling and the Kulldorff's spatial scan statistic (Figure 5).In the most affected areas (clusters), we estimated that 30% of the houses had at least one case and the least affected only 1% (Figure 5).

## Discussion

Our analysis allowed for the identification of the most affected regions in Guinea-Bissau during the 2008 cholera outbreak, and the most affected areas within the capital where 67% of cases were reported. This information was essential for making decisions about where to reinforce treatment and to guide control and prevention activities. As resources are usually limited when responding to cholera outbreaks, knowledge about where to orient interventions is crucial.

Although this analysis provided critical information, this study has limitations. As was the case here, most descriptions of cholera epidemics are *a posteriori*. Comprehensive data collection began one week before the peak of the epidemic and only in the SAB. The description was limited principally due to time and resource constraints as well as the trade-off inherent in emergencies where close concerns and simple analyses are more important than distal and complex analyses [24], [25]. We focused only on one affected area of the city and did not establish statistical associations with environmental, social or cultural risk factors. Strengthening local capacity in surveillance of diseases of epidemic potential remains an ongoing need in countries like Guinea Bissau. Further work should also focus on identifying risk factors that may help orient future interventions. Moreover, we simplified the cluster analysis in the sense that we did not count all the cases in a house; we only classified the households as with or without cases. Thus, the cluster analysis captures the spatial distribution of the risk of primary infections (all houses have at least one) but this can limit the identification of clustering due to factors different from the household location (i.e. secondary transmission at household level).

There are some examples in the literature using spatial techniques to establish associations with environmental variables, but most of these studies are retrospective and come from Bangladesh. Long-term surveillance in Bangladesh, annually affected by cholera, has allowed for research activities regarding the vaccine and etiology of cholera disease. The examples in Africa, where most of the cases occur [4], are scarce and there is a real need for more accurate spatial information. One study in Lusaka, Zambia used a similar methodology to describe the epidemic in one of the most affected neighborhoods of the city [7]. In eastern Democratic Republic of Congo (Kivu provinces) a geographic information system was established, and the authors identified relationships between environmental variables and the number of cholera cases [15]. This analysis allowed the identification of some cities, located on Lake Kivu and Lake Tanganyika, which serve as the main sources of cholera epidemics. Another study in Kumasi, Ghana, analyzed the association of cholera with proximity to refuse dumps [6]. However, in many other countries currently affected by large cholera outbreaks like Zimbabwe, Angola, Mozambique or other west African countries (among those Guinea-Bissau), the spatial epidemiology remains poorly described.

The epidemic prior to 2008 in Guinea-Bissau was in 2005. The same strain (*Vibrio Cholera* O1 El Tor-Ogawa) was circulating during that epidemic and most natural immunity acquired during 2005 had probably vanished during the three-year inter-epidemic period. The AR was higher in 2005 (1.75% vs 0.94%), with a similar CFR. The current outbreak started in May, one month earlier than the outbreak of 2005, but the peak was reached after 22 weeks, thereby doubling the pre-peak period. The first area affected was also different, the current epidemic started in Tombali, but in 2005, the outbreak was first reported in SAB. In both epidemics, the transmission of cholera within SAB facilitated the rapid spread to other regions of the country, and the more affected areas were Bijagos, Biombo and the Sector Autonomo de Bissau in both epidemics.

In all regions, the CFRs were higher at the beginning of the epidemic. This is likely due to the implementation of improved case management and under notification of non-severe cases during the first weeks of the epidemic. Especially high CFRs have been observed in Quinara and Bafata (9.2% and 8.2% respectively), where again the combination of a poor case management and under-notification of non-severe cases likely explain these figures.

The SAB reported 67% of all reported cases. The area with the highest AR was Bandim, playing an important role in the dynamic of the epidemic within the city. Other areas in the southwest of the city such as Quelele, Cuntum, Ajuda or Belem also showed higher ARs than other areas. Within big areas like Bairro Militar, it is likely that the distribution of the AR was not homogeneous with some sub-areas more affected, but we could not test this hypothesis because of the lack of smaller spatial scale population data. We focused our investigation in Bandim because of the high AR and the high percentage of total number of cases reported from this neighborhood. This area is close to the markets and the main road, so it may be important not only because of its disease burden but also because of its potential role in the circulation of cholera in the whole city. Nonetheless, it is important to consider that Bandim has been the site of demographic surveillance system with a focus on infectious diseases. It is possible that the extended presence of these activities in the community leads people to seek treatment promptly, and may therefore account in part for the high attack rate in Bandim [26].

The cluster analysis identified two areas within Bandim at higher risk of finding houses with cases. One was the surroundings of the Caracol market. Different factors potentially explain this higher risk. The first is the market itself; people living in this area are more likely to be in contact with other cholera cases because the market has a large inflow of people. There were also plausible foodborne cases originated in the market, which may disporportionaly affect people living in the surrounding area. Another factor is the large amount of waste around the market and garbage in the streets. Moreover, an open drain passed through the market gathering solid waste and dirty water. Within the market, sanitary conditions were inadequate. The number of latrines was insufficient; there were no hand washing points and the control over the food items sold was insufficient. These factors combined undoubtedly facilitate transmission. As a result of this analysis, the authorities cleaned the market and established washing point and installation of additional latrines. In markets, customers tend to touch, taste and/or smell aliments; this reinforced the need for focused behavioral changes together with sanitation measures around markets.

The other affected area also has a high level of crowding and the confluence of two factors that can increase risk: crowding –this area is crossed by of one of the main streets in Bandim– and an area where runoff accumulates waste. Moreover, the altitude of this zone is almost at sea level. It is likely that the freatic (groundwater) level in this area were higher, which implies less filtration and higher probability of contamination under assumption that the source of drinking water is from local boreholes.

General water and sanitation systems and hygiene condition must be improved to avoid further outbreaks. Nonetheless, these improvements take time and investment and preparedness plans must be developed since outbreaks will continue to occur. Our analysis is useful to orient these plans, and we recommend focusing the preparedness activities in three regions: the Sector Autônomo de Bissau, Biombo and Bijagos, as these areas were the most affected in the 2008 epidemic, and in 2005. The early detection of the outbreak and the control plans are especially important in the capital, Bissau, where most cases and deaths occur. One of the activities that should be planned in advance is the management of the Caracol market and, depending on resources available, the other markets. A cleaning routine should be established, a food safety assessment implemented and latrines and washing points set up. Education and awareness activities are key points to reinforce in order to reduce the impact of future epidemics. These activities are most important in some neighborhoods of the capital: Bandim, Antula, Quelele, Cuntum and Ajuda. Another point to consider among potential control activities are mass vaccination campaigns with the oral cholera vaccine. Feasibility and effectiveness of mass vaccination campaigns in specifically targeted settlements or populations have been demonstrated in endemic areas [27], [28], [29] and their use in targeted locations of Guinea-Bissau, like Bandim, should be considered seriously both as a preventive and a reactive strategy. There is an urgent need to identify new strategies, which are feasible, acceptable and cost-effective to prevent or quickly stop epidemics. Use of oral cholera vaccines might be one of the solutions and its role during outbreaks should be explored.

In conclusion, our study shows the importance of the consideration of space for making decisions about where to reinforce treatment and to guide control and prevention activities in cholera outbreaks. The results of this study also highlight the need for geographical descriptions of cholera epidemics in Africa. Available tools for spatial analysis should be integrated into existing surveillance systems in order to improve preparedness and control of cholera epidemics.
